# Prediction of Diagnosis and Treatment Response in Adolescents With Depression by Using a Smartphone App and Deep Learning Approaches: Usability Study

**DOI:** 10.2196/45991

**Published:** 2023-05-24

**Authors:** Jae Sung Kim, Bohyun Wang, Meelim Kim, Jung Lee, Hyungjun Kim, Danyeul Roh, Kyung Hwa Lee, Soon-Beom Hong, Joon Shik Lim, Jae-Won Kim, Neal Ryan

**Affiliations:** 1 Department of Psychiatry Seoul National University Hospital Seoul Republic of Korea; 2 Department of Computer Science Gachon University Seongnam Republic of Korea; 3 Department of Biomedical Sciences Seoul National University College of Medicine Seoul Republic of Korea; 4 Department of Preventive Medicine Yonsei University College of Medicine Seoul Republic of Korea; 5 Herbert Wertheim School of Public Health and Human Longevity Science University of California San Diego, La Jolla, CA United States; 6 Integrative Care Hub Children's Hospital, Seoul National University Hospital Seoul Republic of Korea; 7 AI.ble Therapeutics Inc Seoul Republic of Korea; 8 Division of Child and Adolescent Psychiatry, Department of Psychiatry Seoul National University College of Medicine Seoul Republic of Korea; 9 Department of Psychiatry University of Pittsburgh Pittsburgh, PA United States

**Keywords:** major depressive disorder, adolescent, deep learning, smart health care, suicide, risk factor, antidepressant treatment, depression, machine learning, smartphone, mobile health, mHealth

## Abstract

**Background:**

Lack of quantifiable biomarkers is a major obstacle in diagnosing and treating depression. In adolescents, increasing suicidality during antidepressant treatment further complicates the problem.

**Objective:**

We sought to evaluate digital biomarkers for the diagnosis and treatment response of depression in adolescents through a newly developed smartphone app.

**Methods:**

We developed the Smart Healthcare System for Teens At Risk for Depression and Suicide app for Android-based smartphones. This app passively collected data reflecting the social and behavioral activities of adolescents, such as their smartphone usage time, physical movement distance, and the number of phone calls and text messages during the study period. Our study consisted of 24 adolescents (mean age 15.4 [SD 1.4] years, 17 girls) with major depressive disorder (MDD) diagnosed with Kiddie Schedule for Affective Disorders and Schizophrenia for School-Age Children-Present and Lifetime Version and 10 healthy controls (mean age 13.8 [SD 0.6] years, 5 girls). After 1 week’s baseline data collection, adolescents with MDD were treated with escitalopram in an 8-week, open-label trial. Participants were monitored for 5 weeks, including the baseline data collection period. Their psychiatric status was measured every week. Depression severity was measured using the Children’s Depression Rating Scale-Revised and Clinical Global Impressions-Severity. The Columbia Suicide Severity Rating Scale was administered in order to assess suicide severity. We applied the deep learning approach for the analysis of the data. Deep neural network was employed for diagnosis classification, and neural network with weighted fuzzy membership functions was used for feature selection.

**Results:**

We could predict the diagnosis of depression with training accuracy of 96.3% and 3-fold validation accuracy of 77%. Of the 24 adolescents with MDD, 10 responded to antidepressant treatments. We predicted the treatment response of adolescents with MDD with training accuracy of 94.2% and 3-fold validation accuracy of 76%. Adolescents with MDD tended to move longer distances and use smartphones for longer periods of time compared to controls. The deep learning analysis showed that smartphone usage time was the most important feature in distinguishing adolescents with MDD from controls. Prominent differences were not observed in the pattern of each feature between the treatment responders and nonresponders. The deep learning analysis revealed that the total length of calls received as the most important feature predicting antidepressant response in adolescents with MDD.

**Conclusions:**

Our smartphone app demonstrated preliminary evidence of predicting diagnosis and treatment response in depressed adolescents. This is the first study to predict the treatment response of adolescents with MDD by examining smartphone-based objective data with deep learning approaches.

## Introduction

One out of 5 adolescents is known to experience major depressive episodes at least once until they reach the adult stage [1]. Major depressive disorder (MDD) is the principal risk factor for suicide, which is the second leading cause of death among adolescents [2]. There are consistent reports on the long-term adverse consequences of adolescent depression, such as impairment in functioning during social and family settings in adulthood [3]. However, recall bias in reporting mood symptoms [4], subjectivity of self-reported symptoms, and the lack of quantifiable markers [5] contribute to the difficulty in treating depression in adolescents. Furthermore, increasing suicidality affects the treatment of depression in adolescents. Previous studies have shown that antidepressant medications may increase the risk of suicidality in adolescents with depression [6]. Suicide rates among treatment nonresponders are observed to be higher than those among treatment responders [7]. Therefore, early diagnosis and treatment of depression are needed. Early prediction of antidepressant treatment response is also of great importance in order to decrease antidepressant-associated suicidality in depressed adolescents.

Newly emerging digital technology presents promising means of dealing with health issues in the field of psychiatry [8]. Monitoring an individual’s status by using smart devices provides promising means of diagnosing and treating adolescents with MDD, given the marked increase and widespread use of smartphones among adolescents. Attempts to predict depressive symptoms based on individual digital phenotype data obtained from smart devices have shown positive results [9-13]. Cao et al [11] used smartphone apps to monitor participants’ mobility and social interactions to predict depressive symptoms in adolescents with an accuracy of 0.88. Ware et al [13] predicted depressive symptoms in young adults with depression with an accuracy of 0.7, based on automatically collected objective smartphone data. However, to the best of our knowledge, only few such studies have focused on depression in the adolescent population [11].

Multiple studies have examined markers for antidepressant treatment response in patients with MDD. Genetic biomarkers have been analyzed using the machine learning approach by the construction of novel predictive models for treatment response [14]. Studies on electrophysiological brain activity using electroencephalogram, functional neuroimaging, and clinical biomarkers have also been conducted [15-19]. However, to our knowledge, no study has assessed antidepressant treatment response by using objective smart device–driven data in populations with MDD. The diagnosis and treatment response of depression have been predicted by machine learning analysis, in particular, by employing deep learning algorithms. Deep learning comprises a set of machine learning algorithms that attempt a high level of abstraction through a combination of nonlinear transformation methods [20]. Deep learning algorithms are superior in processing complex data in which features interact in nonlinear ways, making it especially promising in the field of psychiatric research. Specifically, the deep neural network (DNN) algorithms that we applied in this study are capable of automatic feature extraction, which is the ability to automatically learn higher level representations directly from the data set.

We investigated data obtained from our newly developed smartphone app called Smart Healthcare System for Teens At Risk for Depression and Suicide (STAR-DS), which integrates the passively monitored physical activity and social interaction indices of users. Using deep learning approach in analyzing STAR-DS data, we first aimed to predict the depressive symptoms and diagnosis of depression in adolescents. Second, using STAR-DS data, we aimed to predict the antidepressant treatment response in depressed adolescents.

## Methods

### Ethics Approval

This study was carried out in accordance with the latest version of the Declaration of Helsinki and was approved by the institutional review board of Seoul National University Hospital (1805-008-943). Participants and their legal guardians were provided with detailed information about the study. Written informed consent was obtained from all the participants before study commencement. The smartphone app through which the study data were collected was designed to deidentify the data collected by the study participants.

### Study Participants

In total, 34 adolescents participated in this study; 24 adolescents (mean age 15.4 [SD 1.4] years, 17 girls) with a primary diagnosis of MDD for at least 4 weeks, Children’s Depression Rating Scale-Revised (CDRS-R) [21,22] score ≥40, and Clinical Global Impressions-Severity [23] score ≥4 at baseline evaluation were enrolled from the outpatient clinic of the Division of Child and Adolescent Psychiatry, Seoul National University Hospital. The diagnosis of MDD was based on the Diagnostic and Statistical Manual of Mental Disorders-5 criteria [24], according to the Kiddie-Schedule for Affective Disorders and Schizophrenia for School-Age Children-Present and Lifetime Version [25,26].

Ten nonpsychiatric control participants (mean age 13.8 [SD 0.6] years, 5 girls) without a history of psychiatric disorders were recruited through the Seoul Metropolitan Office of Education and Seoul Metropolitan Community Mental Health Services. The exclusion criteria for both groups included an IQ <70, medical/neurological conditions, and current medications with psychotropic effects other than stimulants for attention-deficit/hyperactivity disorder (ADHD) [27,28]. Participants with first-degree relatives with a history of psychiatric disorders were excluded from the control group. Participants with first-degree relatives with a history of bipolar I disorder were excluded from the MDD group. Moreover, participants with psychotic symptoms, history of psychotic disorder, bipolar disorder, eating disorder, developmental disorder, including autism spectrum disorder, and alcohol or substance abuse within 6 months were excluded from the MDD group.

### Procedures

All study participants were monitored using the STAR-DS app for 5 weeks. After 1 week of baseline monitoring, treatment for the MDD group was initiated with escitalopram in an 8-week open-label trial. The initial dosage of escitalopram was 5 mg/day, which was increased to 10 mg/day after 1 week; thereafter, the dose was flexibly titrated upward to a maximum dose of 25 mg/day for a satisfactory clinical response. The concurrent use of psychotropic medications except for the treatment of ADHD and any psychosocial treatments, including cognitive behavioral therapy, was not allowed during the 8-week antidepressant treatment period. Although stimulant treatment for ADHD was allowed, there was no participant who used stimulants for treatment of ADHD during the study period. The control group was monitored using the STAR-DS app for 5 weeks without any intervention. Adolescents with MDD who had at least a 40% decrease in the CDRS-R total score between the baseline evaluation and the 8th week of treatment were defined as responders [27,29].

### Schedules of Ratings and Assessments

All study participants were assessed using the CDRS-R, Children’s Depression Inventory (CDI) [30,31], Beck Depression Inventory [32,33], Columbia Suicide Severity Rating Scale (C-SSRS) [34,35], Clinical Global Impressions-Severity, Children’s Global Assessment Scale [36], Screen for Child Anxiety Related Emotional Disorders (SCARED) [37,38], Family Adaptability and Cohesion Evaluation Scale-IV (FACES-IV) [39], and IQ at baseline evaluation. A 10-item questionnaire was also administered to assess the smartphone usage pattern of the study participants. All participants were assessed weekly using the CDRS-R, CDI, Beck Depression Inventory, C-SSRS, Clinical Global Impressions-Severity, and Children’s Global Assessment Scale during the STAR-DS monitoring period. Family environment was assessed using the FACES-IV at baseline. The adverse events in the treatment group were assessed using the Side Effects for Children and Adolescents [40] weekly during the STAR-DS monitoring period and biweekly after the termination of STAR-DS monitoring.

### STAR-DS App

STAR-DS is a smartphone app, which was designed only for Android phones, and developed for monitoring the activity and social interaction indices of children and adolescents with depression. The STAR-DS app continues to collect and passively monitor the sensor data without the user’s need to open the app since it is a background app. The collected data are transmitted to the server every 30 minutes and are instantly deleted from the user’s device. Personal identification data were deleted during the storage process, and participants were managed using a novel clinical number as their ID. The app was distributed in the Android package format and was installed on the user’s cellular phone by the research manager. The STAR-DS app can collect data regarding users’ social activity (eg, number of phone calls), physical activity (eg, movement distance), and mobile phone usage status. Moreover, the STAR-DS app provides composite indicators such as text message transmission/reception ratio and sleep volume predictions based on acquired data that allows investigators to understand the various behavioral aspects of the participants.

### Feature Extraction

Each data point was generated at different time scales by the STAR-DS app. Movement distance, amount of activity, and smartphone usage status were real-time monitored. Data regarding phone calls (eg, number, duration), text messages (eg, number of received/sent messages), number of stored phone numbers, and number of image files stored in the device were measured daily. Each feature is described below in detail.

#### Physical Activity

The STAR-DS app traced the participants’ distance moved and rotational momentum by using GPS and a gyroscope in the smartphone. These 2 features are described below in detail.

#### Movement Distance

The app stores the user’s location information every 15 minutes using GPS. The total movement distance was calculated by collating the accumulated location information.

#### Momentum

We used rotational momentum to capture the intensity of action in our participants’ daily lives. It was based on gyroscope data, which measures the rate of rotation using the x, y, and z axes. Data were collected every 15 minutes at 0.1-second interval for 5 seconds; therefore, there were overall 50 coordinates. Transformation of the collected data into scalar quantity was performed using the following formula: √((x2-x1)^2+(y2-y1)^2+(z2-z1)^2); 48 scalar quantities using 50 coordinate values were averaged after excluding the largest and smallest values.

#### Social Activity

We sought to examine the relationship between social activity and depression by using data from phone calls and text messages. Various aspects of phone calls and text messages were analyzed and categorized. The details are described below.

#### Phone Call Data

We collected data on the time, duration, and phone number of every phone call made by the participants. We also examined whether the participant made or received the phone call. The total number and duration of sent/received phone calls made and the number of people contacted by the participants were measured daily.

#### Text Message Data

The time, length (the number of characters), types (messages sent or received), and phone numbers of people contacted by the participants using text messages were logged. The number, length of text messages depending on type, and the number of people the participants exchanged text messages with were extracted daily similar to phone call data analysis. Text message data were collected using the native text messaging app.

#### Smartphone Usage Status

We measured smartphone usage time in addition to the indicators of physical and social activity, which indicated the duration and frequency of smartphone usage. It was measured using real-time monitoring of the on/off status of the device. The number of image files added to the smartphone’s picture files was measured when the user captured a meaningful image or took a photograph of a memorable moment. Hence, we examined the number of image files added on a daily basis as another indicator of social activity.

#### Dosage of the Prescribed Antidepressants

The dosage of escitalopram at each weekly evaluation period and maximum prescribed dose were also included as features for the classification of treatment response and nonresponse groups. Additional features based on statistical values of the classification objects were extracted for the analysis in addition to the abovementioned data obtained directly from the participants’ cellular phones. Three classes of data were used for the deep learning analysis: (1) data collected from smartphone sensors, (2) distance from the mean, and (3) standard deviation of each feature. Data on each feature were averaged daily, and the distance from the weekly mean value was examined, which reflected the daily variation in the features. The standard deviation was measured weekly, which reflected the weekly variation.

### Data Analysis

**Figure 1:**
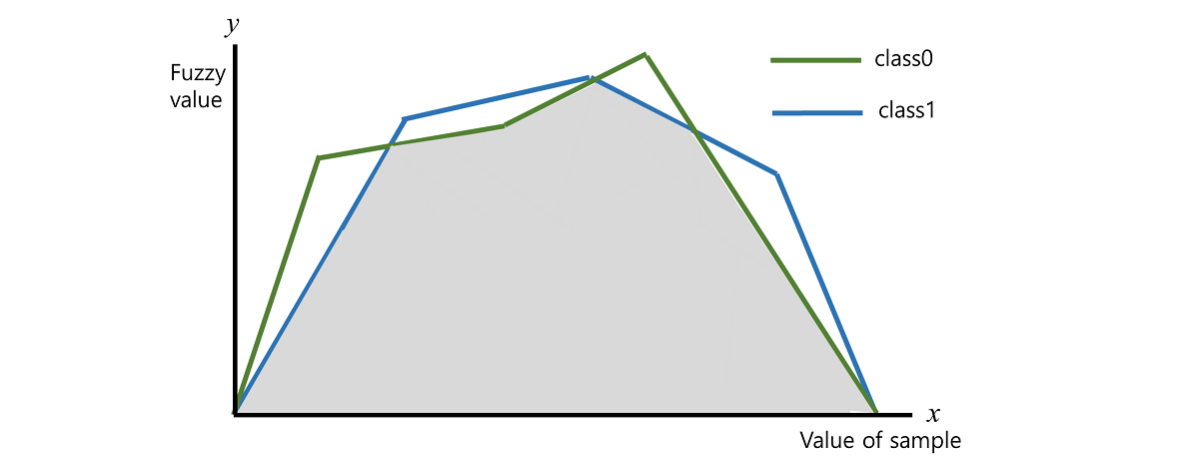
Fuzzy functions distinguishing 2 features.

We used 2-sided Student *t* test for comparing (1) the MDD and control groups and (2) antidepressant treatment responders and nonresponders. Two types of deep learning–based classifications were performed in the main analysis: first, for the classification of the MDD and control groups prior to drug administration and second, to determine the response and nonresponse groups of patients taking medication. In each trial, feature selection was performed simultaneously with classification. We employed the neural network with weighted fuzzy membership functions (NEWFM) algorithm for feature selection and DNN for classification. NEWFM is a machine learning algorithm that applies the neural network learning concept to a fuzzy function, a function based on logic, which makes it possible to quantitatively express ambiguities such as natural languages [41]. In Figure 1, each line represents fuzzy functions for a feature to distinguish between 2 classes. A larger white nonoverlapping area means that the feature can clearly differentiate between the 2 classes. A larger overlapping area means that the difference between the 2 classes measured by the feature is not clear. NEWFM thus uses overlapping and nonoverlapping areas to select features with good classification performance. At every epoch, the rank of the feature is calculated using the overlapping area and the nonoverlapping area of the fuzzy function corresponding to each feature. After deletion of the lowest ranked feature, the feature selection operation is performed by continuously deleting features until the accuracy is no longer improved.

**Figure 2:**
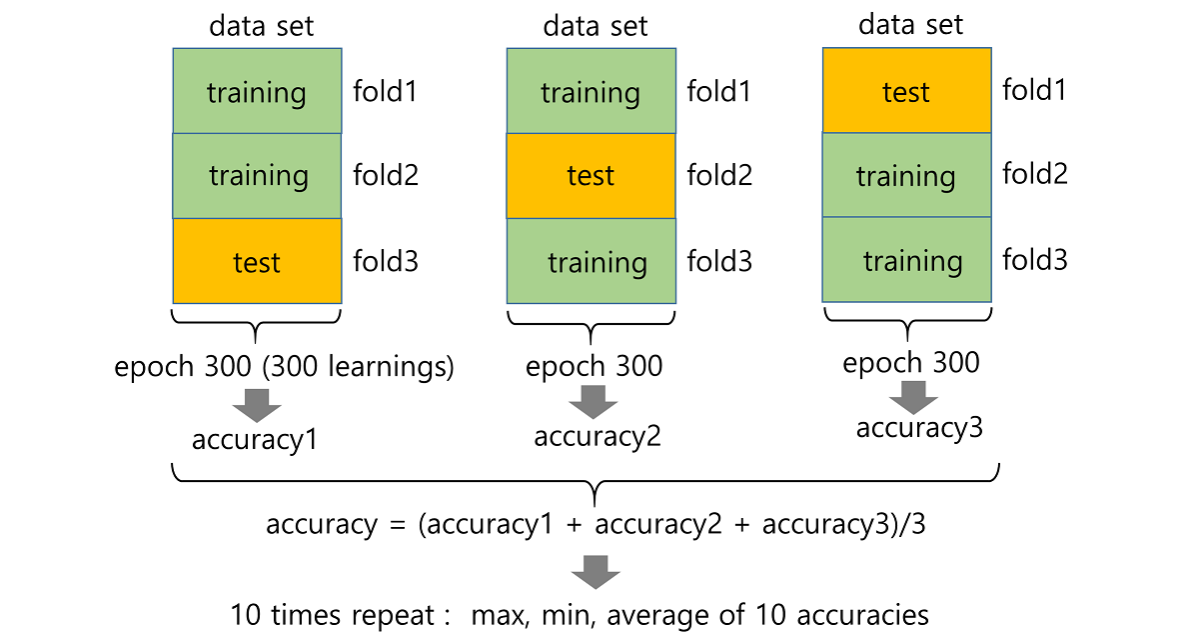
Three-fold validation method used to evaluate the learning model.

The DNN is an artificial neural network with more than one hidden layer between an input layer and output layer, which is a representative deep learning algorithm. The hidden layer is composed of several nodes, and deep learning can be performed by increasing the number of nodes or increasing the hidden layer, and various patterns from input data can be classified. The DNN performs learning by adjusting the weight value of each node through feedforward and backpropagation. In our study, the DNN included 2 hidden layers with 20 nodes per hidden layer. The epoch for each learning was set to 300. A 3-fold validation method was used to evaluate the learning model. The 3-fold cross-validation method splits the data into 3 equal-sized subsets. The model was trained using 2 subsets, leaving one for the validation process as a test subset. The prediction accuracy of the model based on 2 training subsets was measured as training accuracy. After the training was complete, the resulting model was validated using the test subset, assessing the test accuracy. Each subset worked as a test subset alternately (Figure 2).

Three-fold validation of the 300 epochs was performed 10 times. All 300 epochs from each subject were always within the same fold of the 3 folds in each 3-fold validation pass. Three accuracies were produced, and their average was calculated for each 3-fold validation. Therefore, 10 average accuracies and their maximum and minimum values were calculated. We estimated the importance of each feature to examine the relative influence of each feature for predicting the diagnosis of MDD and antidepressant treatment response. We ranked the features in terms of explanatory power while developing the prediction model. We excluded the least important feature and developed a model using the remaining features and repeated the process. We determined the importance of each feature by comparing the averaged ranks of features used in the prediction models. The DNN program was written in Python 3.8 and run on Keras 2.4.3 and Tensorflow 2.3.0. NEWFM was written in Java and run on Windows 10. In order to compare and test the feasibility of our newly adopted method, we also conducted analysis using support vector machine (SVM), a more widely used non–deep learning machine learning algorithm, which was used in previous studies including ours [42-44] as a sensitivity analysis. We used the radial basis function kernel for our SVM classification. For comparison of the distribution of each feature between groups (ie, MDD vs controls and responders vs nonresponders), Fisher exact test was conducted. The SVM program was written in Python 3.8. SVM learning was validated with the 3-fold cross-validation method.

## Results

### Participants’ Demographic and Clinical Characteristics

Two participants dropped out of this study, while the remaining participants completed the study without turning their smartphone off during the study period. The 2 dropouts were due to symptom aggravation and resultant admission. Among 24 participants with MDD, 10 responded to antidepressant treatment. The demographic characteristics of each group are shown in Table 1 and Table 2. The MDD group was significantly older than the control group (*P*<.001). The MDD group showed significantly higher levels of depression and anxiety based on the CDRS-R, CDI, C-SSRS, and SCARED evaluations at baseline than the control group (*P*<.001). The family environment was less favorable in the MDD group (according to the FACES-IV assessment) than that in the control group. A 10-item questionnaire on internet usage patterns showed lower usage of communication apps (eg, Facebook messages) by the MDD group. Age and sex-based differences were not observed between the antidepressant treatment responders and nonresponders. They did not exhibit significant differences in depression and anxiety symptoms at baseline evaluation (Table 2).

**Table 1:** Baseline characteristics of the participants.

			Patient group (n=24)		Control group (n=10)		*P* value
Age (years), mean (SD)			15.4 (1.4)		13.7 (0.7)		<.001
Female, n (%)			17 (71)		5 (50)		.25
**Baseline assessment scores, mean (SD)**							
	CDRS-R^a^ (score)	62.4 (7.0)		27.4 (9.1)		<.001	
	CDI^b^ (score)	37.0 (6.9)		6.8 (7.3)		<.001	
	C-SSRS^c^ (score)	3.6 (1.5)		0.3 (0.9)		<.001	
	SCARED^d^ (score)	45.0 (17.6)		12.7 (12.4)		<.001	
	IQ (points)	104.9 (16.6)		111.6 (6.1)		.09	
	FACES-IV^e^ (score)	41.8 (12.5)		59.6 (12.7)		.001	

^a^CDRS-R: Children’s Depression Rating Scale-Revised.

^b^CDI: Children’s Depression Inventory.

^c^C-SSRS: Columbia Suicide Severity Rating Scale.

^d^SCARED: Screen for Child Anxiety Related Emotional Disorders.

^e^FACES-IV: Family Adaptability and Cohesion Evaluation Scale-IV.

**Table 2:** Baseline characteristics of the antidepressant treatment responders and nonresponders.

			Responder group (n=10)		Nonresponder group (n=14)		*P* value
Age (years), mean (SD)			15.8 (1.2)		15.1 (1.5)		.26
Female, n (%)			7 (70)		10 (71)		.94
**Baseline assessment scores, mean (SD)**							
	CDRS-R^a^ (score)	60.4 (5.4)		63.9 (7.7)		.24	
	CDI^b^ (score)	36.8 (6.0)		37.2 (7.8)		.89	
	C-SSRS^c^ (score)	4.1 (0.9)		3.2 (1.8)		.12	
	SCARED^d^ (score)	41.6 (15.6)		47.4 (19.1)		.44	
	IQ (points)	109.9 (19.7)		101.3 (13.6)		.22	
	FACES-IV^e^ (score)	40.8 (12.6)		42.4 (12.8)		.76	

^a^CDRS-R: Children’s Depression Rating Scale-Revised.

^b^CDI: Children’s Depression Inventory.

^c^C-SSRS: Columbia Suicide Severity Rating Scale.

^d^SCARED: Screen for Child Anxiety Related Emotional Disorders.

^e^FACES-IV: Family Adaptability and Cohesion Evaluation Scale-IV.

### Prediction of MDD and Antidepressant Treatment Response by Using Deep Learning

Features collected using the STAR-DS app were randomly sampled from each class to be used as test samples in the 3-fold validation process. After collating and including all 3 data sets, raw data, distance from the mean, and standard deviation, deep learning showed 96.3% training accuracy and 77% 3-fold average accuracy for predicting MDD. Deep learning showed 94.2% training accuracy and 76% 3-fold average accuracy for predicting the treatment response in the MDD group. The accuracy of SVM in predicting MDD was 93.4% in training and 75% in 3-fold average, respectively. SVM predicted treatment response in the MDD group with 99.2% training accuracy and 85.1% 3-fold average accuracy.

### Comparison of Features Between the MDD and Control Groups and Between Treatment Response and Nonresponse Groups

The distribution pattern of the participants according to the value of the representative features is presented in Multimedia Appendix 1. The MDD group used their smartphones for longer periods of time. Moreover, the MDD group showed a tendency to receive more phone calls compared to the control group. Regarding the number of text messages received, the 2 groups showed similar distribution patterns. The MDD group tended to move longer distances. Prominent differences were not observed in the pattern of each feature of the treatment responder and nonresponder groups.

### Feature Importance Using the Deep Learning Method

We ranked the importance of features in predicting each diagnosis of depression and antidepressant treatment response by using the deep learning method. The duration of smartphone use ranked the highest for predicting MDD in adolescents (Table 3). The total duration of the calls made and number of calls received (both call-related features) ranked second and third, respectively. The total time of calls received was ranked first in the prediction of treatment response. Movement distance, which ranked fourth in the prediction of the diagnosis, ranked second in response prediction. The duration of smartphone use, which ranked first in prediction of diagnosis, ranked 12th in treatment response prediction.

**Table 3:** Feature importance according to the average rank of each feature in predicting depression.

Rank	MDD^a^ group versus control group	Treatment responder group versus nonresponder group
1	Screen usage duration	Total time of calls received
2	Total time of calls made	Movement distance
3	Number of calls received	Number of people called
4	Movement distance	Number of calls sent
5	Momentum	Maximum dose of medication
6	Number of people messaged	Number of text messages received
7	Number of text messages received	Total time of calls made
8	Total time of calls received	Number of messages sent
9	Total length of messages received	Number of calls received
10	Number of calls sent	Added image files
11	Total length of messages sent	Total length of messages received
12	Number of messages sent	Screen usage duration
13	Number of people called	Momentum
14	Added image files	Total length of messages sent
15	N/A^b^	Number of people messaged
16	N/A	Dosage of medication

^a^Major depressive disorder.

^b^N/A: not applicable.

## Discussion

### Principal Findings

This study is the first to examine the feasibility of predicting the diagnosis of MDD and antidepressant treatment response in an adolescent population by using the STAR-DS smartphone app. Adolescents showed high adherence rate to smartphone use with the installed STAR-DS app. Employing the deep learning approach, we predicted the diagnosis of MDD and antidepressant treatment response with a relatively favorable accuracy. Through prediction models, we examined the significance of each feature in predicting the diagnosis and treatment response. The test accuracy of 0.77 in predicting the diagnosis of MDD obtained in this study is similar to that reported by other studies [12,13] that used non–deep learning machine learning analysis to predict mood symptoms with objective data acquired using digital devices. Ware et al [13] predicted the depressive mood of 182 college students with an accuracy of 0.7. Tazawa et al [12] reported an accuracy of 0.76 in predicting depressive symptoms of 86 adults. Importantly, the test accuracy was 0.76 in predicting antidepressant treatment response. This was the first prediction result of antidepressant treatment response based on smart device–driven objective data. Moreover, sensitivity analysis adopting SVM showed similar training and 3-fold average accuracy in predicting MDD and antidepressant treatment response. These results seem promising, considering the small sample size of our study. Deep learning analysis, which we adopted, has relative strength in dealing with larger sets of data. Having achieved comparable results with SVM in the small sample size of our pilot study, we expect enhanced accuracy of deep learning with larger data, which would be collected in future research.

The comparison of the MDD group and control group in our study revealed some differences in several monitoring features. First, adolescents with MDD tended to receive more calls compared to the control participants. Previous studies using clinical samples have confirmed this result [9]. Faurholt-Jepsen et al [45] reported a significant association between incoming calls and depressive symptoms in their study with 29 adults. This may be because patients with MDD probably received more attention from their family members, friends, or acquaintances owing to the status of their health, considering that the MDD group had a less favorable baseline family environment compared to the controls. Second, adolescents with MDD showed an inclination to spend more time on their smartphones, which is in line with the results of a previous study [9]. Thus, we can infer that adolescents with MDD spent more time using smartphones but did not use it for social communication, as indicated by the differences in the smartphone usage patterns between the MDD and control groups. Third, the MDD group tended to move longer distances than the control group. This is in contrast with previous study results, which tended to report negative correlations between longer movement distance and depressive symptom severity [9]. We assume that such differences might result from differences in study samples since previous study results were mainly based on nonclinical samples of adults. Longer movement distance of depressed participants might reflect heterogeneity in the symptomatic features of adolescent depression [46].

We could not find prominent differences between each feature of the antidepressant treatment response and nonresponse groups. Although the underlying pathophysiology of both subtypes of depression might differ, the behavioral manifestation of the depressive symptoms seemed to be similar in one-to-one comparison of each feature. However, the app of the deep learning approach facilitated the differentiation between the 2 groups with 76% accuracy. Such a result implies the feasibility of deep learning approach in classification, which is difficult to be achieved through non–machine learning methods, and therefore, there is a further need for deep learning approaches. The results of our deep learning analysis revealed that the duration of smartphone usage was the most important feature for predicting the diagnosis of MDD. This is in line with the results of previous studies, which have also reported fairly consistent results regarding the association between longer smartphone use and depressive symptoms [9,47]. It is also noteworthy that the significant features that predicted MDD or treatment response were different in this study. Based on our results, smartphone usage duration, which ranked first in predicting MDD, ranked 12th in the prediction of treatment response. Moreover, the total duration of the calls received ranked first for predicting the treatment response, while it ranked 8th for predicting MDD. This may mean that smartphone usage duration is a prevalent phenotype of depression in treatment responsive and nonresponsive depression, unlike other behavioral or social features of depression, such as movement distance, which ranked high in both analyses, making it difficult to distinguish between the 2 groups by using this feature. In contrast, the total duration of the received calls could successfully distinguish between treatment responsive depression from treatment nonresponsive depression but may be a relatively prevalent feature in participants with MDD and nonpsychiatric participants, making it difficult to predict the diagnosis of MDD. Such results suggest that the requisite features should be differentiated according to the purpose of the prediction.

### Limitations

This study has several limitations. First, our study protocol was different from the treatment guideline for adolescent depression, which recommends an app of cognitive behavioral therapy alone or in combination with pharmacotherapy [48]. This deviation was due to our intention to focus on predicting antidepressant treatment response, which we thought would have more clinical importance relative to cognitive behavioral therapy, a treatment modality reported to be generally tolerable regarding its side effects. Such an approach is effective in clearly showing the association between smartphone-measured objective data and the antidepressant treatment response. However, it carries the risk of lacking generalizability in that it was different from the recommended real-world treatment guidelines.

Second, the control group was significantly younger than the patient group for the prediction of MDD diagnosis (*P*<.001). Although there are no published studies on the differences in the symptomatology of adolescent depression according to age, the possibility of age’s differential effect on the study outcome such as depression and responder status should be considered. Moreover, age difference might correspond to behavioral differences leading to differences in the independent variables. Further studies with more data of age-matched participants are needed.

Third, deep learning analysis of smartphone data showed high training accuracy, but the test accuracy was relatively low for predicting the depression and antidepressant treatment response. These outcomes can be attributed mainly to the small sample size in this study. Separating the training and testing data and thereby enhancing the accuracy of model was not adoptable due to the limitations in the sample size. Combined with the high feature-to-sample ratio, this might have led to overfitting of the model [49]. The general sample size required for training of the machine learning models using DNNs is uncertain [50]. However, existing literature suggests that deep learning might not show superior performances in comparison to other machine learning methods, including SVM, for sample sizes <1000 [51,52]. Furthermore, previous evaluation adopting similar models used in this study has shown test accuracy dramatically increasing until the sample reaches 1000 and then levelling off. In line with previous studies, we expect further studies with larger samples, based on our pilot study, will be able to present enhanced accuracy of analyzing smartphone data.

Fourth, some categories (eg, the number of calls received) used for the prediction of MDD diagnosis and antidepressant response provided limited variance. The modal value of the number of calls received was 1, a value small enough to be affected by a single event. This might have affected the results of this study.

Fifth, there were challenges using smartphone data for research. Social activity indices measured using phone calls and text messages may not accurately reflect real social interactions, since smartphone users commonly use instant messaging apps for communication. Further, there is possibility that differences in smartphone usage might not accurately reflect behavioral differences due to overall high smartphone reliance in the adolescent population. Moreover, STAR-DS is an Android-only app. Including only Android users may limit the generalizability of our results, considering previous studies reporting demographic and personality differences between Android and iPhone users [53].

## Appendix

Multimedia Appendix 1 Distribution pattern of the participants according to the value of the representative features.

## Abbreviations

ADHD: attention-deficit/hyperactivity disorder
CDI: Children’s Depression Inventory
CDRS-R: Children’s Depression Rating Scale-Revised
C-SSRS: Columbia Suicide Severity Rating Scale
DNN: deep neural network
FACES-IV: Family Adaptability and Cohesion Evaluation Scale-IV
MDD: major depressive disorder
NEWFM: neural network with weighted fuzzy membership functions
SCARED: Screen for Child Anxiety Related Emotional Disorders
STAR-DS: Smart Healthcare System for Teens At Risk for Depression and Suicide
SVM: support vector machine

## Data Availability

Data from this study are available from the corresponding author upon request.
