# Supplementary material for: Prediction of Diagnosis and Treatment Response in Adolescents With Depression by Using a Smartphone App and Deep Learning Approaches: Usability Study
Source: JMIR Form Res. 2023 May 24;7:e45991. doi: 10.2196/45991 (PMC10248781; doi:10.2196/45991)

**Multimedia Appendix 1**

**Figure S1:** [Distribution of features between MDD and control group.](https://formative.jmir.org/api/download?filename=0eeced82534e871880d7d656cfd2ab8d.png&alt_name=45991-747752-1-SP.png)


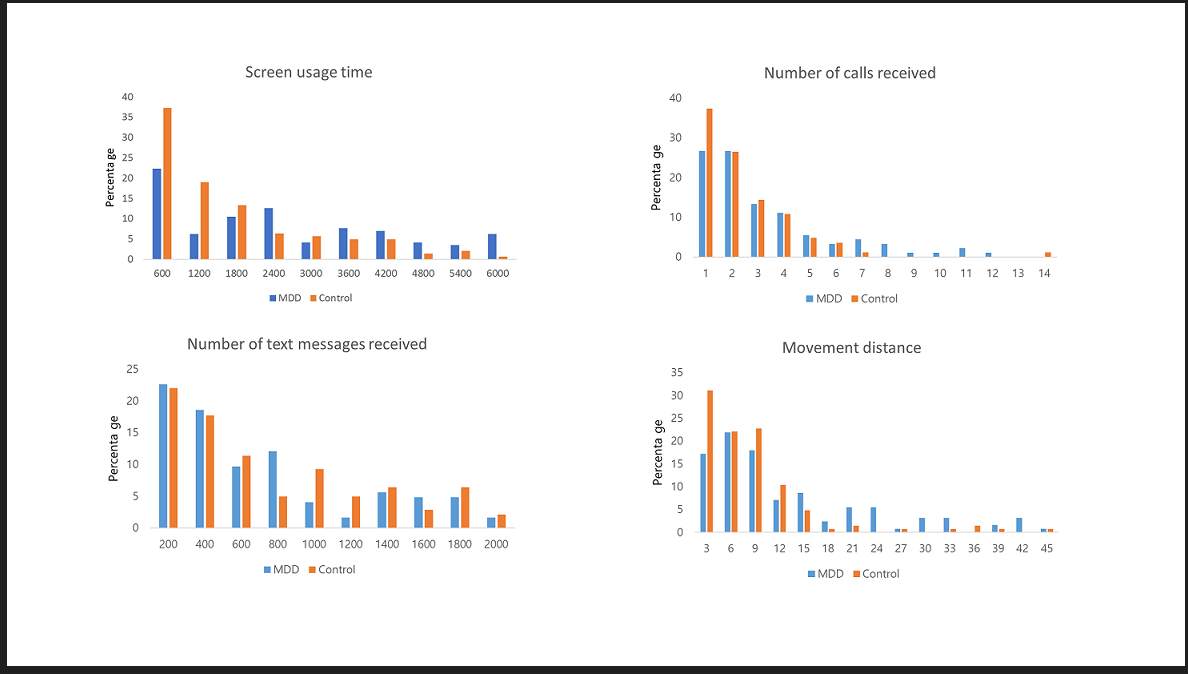


**Figure S2:** [Distribution of features between treatment response and non-response group.](https://formative.jmir.org/api/download?filename=b6b8d90f7de7e089384407e5666a2633.png&alt_name=45991-747754-1-SP.png)


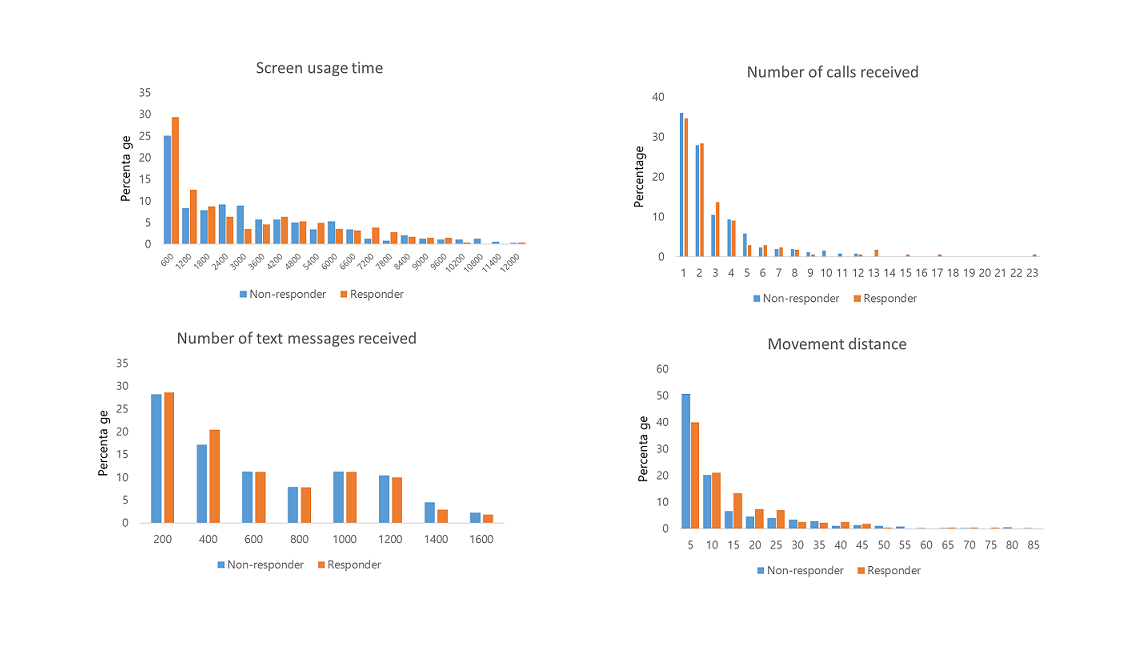

Supplement: Multimedia Appendix 1 [file formative_v7i1e45991_app1.docx]
